# Hybrid Imaging of *Aspergillus fumigatus* Pulmonary Infection with Fluorescent, ^68^Ga-Labelled Siderophores

**DOI:** 10.3390/biom10020168

**Published:** 2020-01-22

**Authors:** Joachim Pfister, Dominik Summer, Milos Petrik, Marta Khoylou, Alexander Lichius, Piriya Kaeopookum, Laurin Kochinke, Thomas Orasch, Hubertus Haas, Clemens Decristoforo

**Affiliations:** 1 Department of Nuclear Medicine, Medical University Innsbruck, A-6020 Innsbruck, Austria; joachim.pfister@i-med.ac.at (J.P.); summer.dominik@gmail.com (D.S.); gamsuk@hotmail.com (P.K.); Laurin.Kochinke@student.i-med.ac.at (L.K.); 2 Institute of Molecular and Translational Medicine, Faculty of Medicine and Dentistry, Palacky University Olomouc, 772-00 Olomouc, Czech Republic; MilosPetrik@seznam.cz (M.P.); m.khoylou@seznam.cz (M.K.); 3 Department of Microbiology, University Innsbruck, A-6020 Innsbruck, Austria; alexander.lichius@uibk.ac.at; 4 Division of Molecular Biology, Medical University Innsbruck, A-6020 Innsbruck, Austria; Thomas.Orasch@hki-jena.de (T.O.); hubertus.haas@i-med.ac.at (H.H.)

**Keywords:** invasive pulmonary aspergillosis, siderophores, fluorescence microscopy, PET, near infrared, gallium-68

## Abstract

*Aspergillus fumigatus* (*A. fumigatus*) is a human pathogen causing severe invasive fungal infections, lacking sensitive and selective diagnostic tools. *A. fumigatus* secretes the siderophore desferri-triacetylfusarinine C (TAFC) to acquire iron from the human host. TAFC can be labelled with gallium-68 to perform positron emission tomography (PET/CT) scans. Here, we aimed to chemically modify TAFC with fluorescent dyes to combine PET/CT with optical imaging for hybrid imaging applications. Starting from ferric diacetylfusarinine C ([Fe]DAFC), different fluorescent dyes were conjugated (Cy5, SulfoCy5, SulfoCy7, IRDye 800CW, ATTO700) and labelled with gallium-68 for in vitro and in vivo characterisation. Uptake assays, growth assays and live-cell imaging as well as biodistribution, PET/CT and ex vivo optical imaging in an infection model was performed. Novel fluorophore conjugates were recognized by the fungal TAFC transporter MirB and could be utilized as iron source. Fluorescence microscopy showed partial accumulation into hyphae. µPET/CT scans of an invasive pulmonary aspergillosis (IPA) rat model revealed diverse biodistribution patterns for each fluorophore. [^68^Ga]Ga-DAFC-Cy5/SufloCy7 and -IRDye 800CW lead to a visualization of the infected region of the lung. Optical imaging of ex vivo lungs corresponded to PET images with high contrast of infection versus non-infected areas. Although fluorophores had a decisive influence on targeting and pharmacokinetics, these siderophores have potential as a hybrid imaging compounds combining PET/CT with optical imaging applications.

## 1. Introduction

Fungal infections of humans are widespread and can appear in many different forms. Infections of the skin and nails are very common, like onychomycosis or oral thrush [1]. Most people will suffer from those at least once in their lifetime, but these infections are easily diagnosed and respectively curable. In contrast, severe systemic fungal infections kill about one and a half million people every year [2]. One of the most prominent examples is invasive pulmonary aspergillosis (IPA), an opportunistic infection of the lung mainly caused by *Aspergillus fumigatus* (*A. fumigatus*). Worldwide, an estimate of over 200,000 patients are affected, with a lethality rate of 30–95% [2]. The vegetatively produced spores (conidia), *A. fumigatus* uses for its own distribution, are the prominent infectious agent in air samples, including air from hospitals. Up to 1000 conidia are inhaled each day, which, due to their small size of 2–4 µm, can even reach the alveoli in the lungs [3]. The cilia of epithelia cells easily clear the airway of these spores and macrophages in the alveola eliminate invading microorganisms. However, in case the immune system is suppressed including genetic reasons, medication (e.g., chemotherapy, after organ transplantation and concomitant immunosuppressive drug therapy) or deficient lung functions due to comorbidity (e.g., COPD or mechanical ventilation in intensive care units), the conidia can germinate, invade the lung tissue and develop IPA. One reason for the high lethality rates of IPA originate from insufficient diagnostic methods. Even if IPA is diagnosed early and treated correctly, an overall mortality of 50% still occurs. However, when diagnosis is missed or delayed, IPA is nearly 100% fatal [2]. Standard procedures like computed tomography scans, culture of sputum/bronchoalveolar lavage (BAL) samples or galactomannan test in serum/BAL are currently available but slow and most often of poor sensitivity and/or specificity [4]. Therefore, new non-invasive methods to diagnose the early onset of IPA are urgently needed.

This approach exploits the special survival strategy *A. fumigatus* utilizes in the human host. The human lung is generally a very hostile environment for most filamentous fungus: high temperature, high CO_2_ concentration, neutral to basic pH, high phagocytic activity and large scale secretion of mucins and surfactant proteins prevent fungal germination [5,6]. Conidia of *A. fumigatus*, however, tolerate these adverse conditions, germinate and finally invade the bloodstream with hyphae to take up nutrients. One of the most important limiting factors of conidial development in the human host is iron supply [7]. In the human bloodstream, iron is bound to transferrin or in the erythrocytes as haemoglobin. In addition, under pathogenic conditions, enhanced labile plasma iron can be found, but they are not directly available for the pathogen [8,9]. To circumvent this, *A. fumigatus* produces siderophores, small organic molecules with very high affinity for ferric ions, which are released into the infected tissue to trans-chelate iron. Siderophore bound iron is subsequently re-absorbed by specific siderophore transporters (SIT) [10]. This iron acquiring system is essential for the virulence of the fungal pathogen, highly upregulated during infection and not found in the human body [11]. Therefore, SITs serve as a perfect target for diagnostic applications.

**Figure 1:**
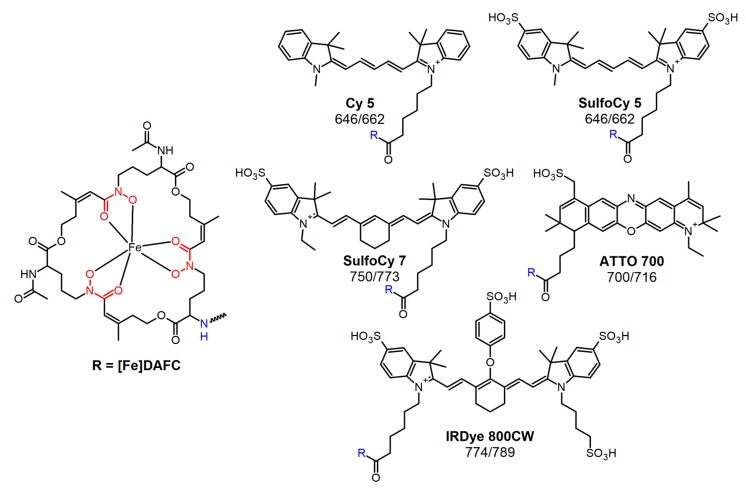
Chemical structure of diacetylfusarinine C (DAFC) and conjugated fluorophores with their corresponding absorption/emission [nm].

Different types of siderophores are found in *A. fumigatus*: desferri-fusarinine C (FsC) and desferri-triacetylfusarinine C (TAFC) which are secreted for iron acquisition as well as desferri-ferricrocin (FC) and desferri-hydroxyferricrocin, which are siderophores employed for internal transfer and storage of iron [12]. Uptake of external siderophores is managed by different transporters. *A. fumigatus* uses the MirB transporter for [Fe]TAFC uptake [13]. TAFC and FsC consist of three N5-cis-anhydromevalonyl-N5-hydroxy-L-ornithine units which are linked by ester bonds to a cyclic molecule (Figure 1). The three amino groups are either acetylated (TAFC) or present as free amines (FsC), that allow chemical modification [14]. A high iron binding affinity of pM = 31.8 enables TAFC trans-chelation of ferric irons from transferrin (pM = 23.6) in the human host [15]. This high ferric iron affinity can also be used to exchange iron by gallium-68, which has the same size and charge as ferric iron, and facilitates the application of [^68^Ga]Ga-TAFC as a radioactive tracer for the rapid localisation of IPA infections by PET/CT scan [16]. Moreover gallium-68 can be obtained from a ^68^Ge/^68^Ga-generator that is commercially available and easy to handle.

Therapeutic intervention is also highly challenging due to an increasing resistance of *A. fumigatus* to commonly used antifungals, and the rapid decline of the patient’s health condition. Surgical procedures may be required to remove affected lung tissue [17]. Image guidance may help to visualize infected lung tissue intraoperatively. Chemical modification by introducing fluorophores into TAFC structure can provide novel hybrid imaging agents. TAFC can be in parallel radiolabelled with gallium-68 for PET diagnostic purposes as well as conjugated to a fluorophore for optically guided surgery, which has been successfully demonstrated for oncological applications [18,19,20]. A major challenge is to find the optimal fluorophore, which provides suitable pharmacokinetic properties and the correct wavelength [21]. The ideal detection window is between 650–850 nm, where the surrounding human tissue has a low intrinsic autofluorescence.

In this paper, we describe the chemical modifications of TAFC with different near infrared (NIR) dyes and the evaluation of their pharmacological properties. We also investigate possible hybrid imaging applications in vivo by using an IPA rat model with a µPET/CT and optical imaging system [16].

## 2. Materials and Methods

### 2.1. Chemicals

All chemicals were purchased from commercial sources as reagent grade and used without further purification unless otherwise stated. Fluorescent dyes were obtained with carboxylic coupling site and used for synthesis without further purification: Cyanine5 carboxylic acid (Cy5); sulfo-Cyanine5 carboxylic acid (SulfoCy5); sulfo-Cyanine7 carboxylic acid (SulfoCy7) (Lumiprobe GmbH, Hannover, Germany). IRDye^®^ 800CW carboxylic acid (IR-Dye 800CW) (LI-COR Biotechnology, Lincoln, NE, USA). ATTO 700 carboxylic acid (ATTO 700) (ATTO-TEC GmbH, Siegen, Germany) (Figure 1).

### 2.2. Synthesis of Fluorophore Conjugates

[Fe]FsC was used as a starting material to produce [Fe]DAFC as previously described [22]. All fluorescent dyes were used as carboxylic acid derivatives and activated with O-(7-azabenzotriazol-1-yl)-1,1,3,3-tetramethyluronium-hexafluorophosphat (HATU) for conjugation to the free amine of [Fe]DAFC in DMF. After reaction (<2 h) at ambient temperature and under light exclusion, quantitative conjugation was reached and the reaction solution was purified by preparative RP-HPLC to give a coloured solid powder after lyophilisation. Identity was confirmed by MALDI-TOF MS. For detailed chemical information, see supplementary material.

For radiolabelling, iron was removed from the complex by incubation with a 1000-fold excess of ethylenediaminetetraacetic acid (EDTA) at pH 4 and subsequent purification by preparative RP-HPLC as previously described [23].

### 2.3. Radiolabelling

Fractionated elution of ^68^Ge/^68^Ga-generator (IGG100. Eckert & Ziegler Isotope Products, Berlin, Germany; nominal activity of 1850 MBq) with 0.1 M hydrochloric acid (HCL, Rotem Industries, Arva, Israel) was used to obtain ^68^GaCl3 (~250 MBq) in 1 mL eluate. For labelling, 10 µg (5–8 nmol) of DAFC-conjugate were mixed with 200 µL gallium eluate (~15–30 MBq) and the pH was adjusted to 4.5 by adding 20 µL of sodium acetate solution (1.14 M) per 100 µL eluate. The mixture was left to react for 10 min at RT and finally analyzed by radio-TLC and radio-RP-HPLC [23].

### 2.4. In Vitro Characterisation

#### 2.4.1. Distribution Coefficient (Log D)

Log D was determined by measuring the distribution of each compound between octanol and PBS buffer. For this purpose, radiolabelled compound was dissolved with PBS to 1 mL (~9 µM). Aliquots of 50 µL were added to 450 µL PBS and 500 µL octanol, shaken for 20 min with 1400 rpm at RT (MS 3 basic vortexer, IKA, Staufen, Germany) followed by centrifugation for 2 min at 4500 rpm (Eppendorf Centrifuge 5424, Eppendorf AG, Hamburg, Germany). Hereafter, 200 µL of each phase were collected and measured in a 2480 automatic Gamma counter Wizard 2 3″ (PerkinElmer, Waltham, MA, USA). Log D value was calculated using Excel. (*n* = 3, six technical replicates)

#### 2.4.2. Protein Binding

Radiolabelled compound was diluted with PBS to 1 mL (~9 µM) and 50 µL of that solution was added to 450 µL serum or 450 µL PBS as a control. After 30, 60 and 120 min incubation at 37 °C, aliquots of 25 µL were analysed by size exclusion chromatography using MicroSpin G-50 columns (Sephadex G-50, GE Healthcare, Vienna, Austria) according to the manufacturer’s protocol. Hereafter, column and eluate were measured separately in the gamma counter to calculate percentage of free- (column bound) and protein-bound (eluate) fraction.

#### 2.4.3. Uptake and Competition Assay

Uptake assays were performed as previously published [14]. Briefly, 180 µL of *A. fumigatus* culture in iron-depleted and iron-replete media were added in 96-well MultiScreen Filter Plates HTS (1 μm glass fiber filter, Merck Millipore, Darmstadt, Germany) and pre-incubated for 15 min with either PBS or [Fe]TAFC (blocking solution). Subsequently, radiolabelled compound (final concentration approximately 90 nM) was added and incubation continued for 45 min at room temperature. Dry filters were measured in the gamma counter.

Competition assays were performed in the same way with slight modifications. Fungal cultures were pre-incubated with iron-labelled fluorophore conjugates for 15 min and the uptake value of [^68^Ga]Ga-TAFC into hyphae was determined after another 45 min of incubation.

#### 2.4.4. Growth Promotion Assay

To investigate the ability of *A. fumigatus* to use iron containing siderophores for its metabolism, a mutant strain (ΔsidA/ΔftrA) lacking two genes, sidA and ftrA was point inoculated (10^4^ conidia) in 24-well plates, containing 0.5 mL of aspergillus minimal media agar with [Fe]siderophore concentrations ranging from 0.1–50 µM. Plates were incubated for 48 h at 37 °C in a humidity chamber and visually assessed afterwards [14].

#### 2.4.5. Fluorescence Microscopy

Fluorescence microscopy was performed for [Fe]DAFC-Cy5 and -SulfoCy5 allowing an excitation wavelength below 700 nm. Fungal cultures were prepared in µ-Slide 8 Well chambered coverslips (ibidi GmbH, Martinsried, Germany). Each well was inoculated with 5000 Spores in 200 µL of minimal medium and incubated at 37 °C in a humidified chamber. *A. fumigatus* (ATCC 46645) was cultivated for 14 h and *A. terreus* (ATCC 3633) for 48 h to obtain well developed germlings and young hyphae without extensive germling or vegetative hyphal fusion. For microscopy, a Leica TCS SP5 II inverted confocal laser scanning microscope was used with HeNe laser (10 mW: 633 nm) as excitation light source: AOBS (Acousto-Optical Beam Splitter) (640–690 nm) and Leica HyD detector. Fluorophores were used at a final concentration of 2.5 µM and incubated for 10–40 min. Excitation laser intensity during imaging was kept to a minimum to reduce photobleaching and phototoxic effects to the cells while still achieving good signal-to-noise ratios. Images were recorded with a maximum resolution of 1024 × 1024 pixels and saved as TIFF. Some images represent a z-Stack that is labelled in the particular picture description. Apart from brightness adjustments and cropping using the ImageJ 1.52a opensource software platform (Wayne Rasband, NIH, Bethesda, MD, USA), images were not subjected to further manipulation.

### 2.5. In Vivo Characterisation

All animal experiments were conducted in compliance with the Austrian and Czech animal protection laws and with approval of the Austrian Ministry of Science (BMWFW-66.011/0161-WF/V/3b/2016), the Czech Ministry of Education Youth and Sports (MSMT-21275/2016-2) and the institutional Animal Welfare Committee of the Faculty of Medicine and Dentistry of Palacky University in Olomouc.

#### 2.5.1. Invasive Pulmonary Aspergillosis Model in Rats

Female Lewis rats of 2–3 months age were treated with the immunosuppressant cyclophosphamide (Endoxan, Bayter, 75 mg/kg i.p.) 5 days and 1 day before *A. fumigatus* inoculation to induce neutropenia. To avoid bacterial superinfections, animals repeatedly (5 days, 1 day before and on the day of inoculation) received antibiotic teicoplanin (Targocid, Sanofi, 35 mg/kg–5 days before i.m. or 25 mg/kg i.m.–1 day before and on the day of inoculation) and additional antibiotics were administered by drinking water (Ciprofloxacin, 2 mM, Polymyxin E, Colomycin, 0.1 mM) for the duration of the experiment. Fungal infection was established by intratracheal application of 100 µL of *A. fumigatus* spores (10^9^ CFU/mL *A. fumigatus* 1059 CCF) using TELE PACK VET X LED system equipped with a flexible endoscope (Karl Stroz GmbH & Co. KG, Tuttlingen, Germany) only [24].

#### 2.5.2. Micro PET/CT and Optical Imaging

In vivo PET/CT images were acquired with an Albira PET/SPECT/CT small animal imaging system (Bruker Biospin Corporation, Woodbridge, CT, USA). Female Lewis rats were retro-orbitally (r.o.) injected with radiolabelled fluorophore conjugate in a dose of 5–10 MBq corresponding to ~2 μg of DAFC-conjugate per rat. Animals were anaesthetized with isoflurane (Forene^®^, Abbott Laboratories, Abbott Park, IL, USA) (2% flow rate) and positioned prone head first in the Albira system before the start of imaging. Static PET/CT imaging was carried out 45 min p.i. for all tested compounds. PET/CT imaging of infected animals was performed three days after the inoculation with *A. fumigatus* spores. A 10-min PET scan (axial FOV 148 mm) was performed, followed by a triple CT scan (axial FOV 3 × 65 mm, 45 kVp, 400 μA, at 400 projections). Scans were reconstructed with the Albira software (Bruker Biospin Corporation, Woodbridge, CT, USA) using the maximum likelihood expectation maximization (MLEM) and filtered backprojection (FBP) algorithms. After reconstruction, acquired data were viewed and analysed with PMOD software (PMOD Technologies Ltd., Zurich, Switzerland). 3D images were obtained using VolView software (Kitware, Clifton Park, NY, USA). After PET/CT imaging, the animals were sacrificed by exsanguination and lungs were collected for ex vivo fluorescence imaging.

Fluorescence imaging of excised lungs was performed with an in vivo MS FX PRO small animal imaging system (Bruker Biospin Corporation, Woodbridge, CT, USA) 1.5 h p.i. An appropriate filter set (excitation = 630 nm, emission = 700 nm for Cy5, SulfoCy5, ATTO 700 and TAFC, and excitation = 700 nm, emission = 790 nm for SulfoCy7, IRDye 800CW and TAFC) was used for acquiring the fluorescence images of DAFC-conugates. Identical illumination settings (acquisition time = 30 s, filters = 630/700 or 700/790 nm, f-stop = 2.8, field of view = 100 mm and binning = 2 × 2) were used for acquiring all images, and fluorescence emission was normalized to photons/s/mm^2^. Images were acquired and analysed using Bruker MI SE software (Bruker Biospin Corporation, Woodbridge, CT, USA).

## 3. Results

### 3.1. Synthesis of Fluorophore Conjugates

Fluorescent dye coupling was straightforward with reaction times under 2 h. Yields for iron-containing and iron-free compounds varied between 60–80% with a high chemical purity of >95%.

### 3.2. Radiolabelling

The mild labelling conditions at room temperature and short time of 10 min allowed high radiochemical yields for all compounds, respectively. Both radio-ITLC and radio-RP-HPLC showed radiochemical purity over >95%. (representative HPLC radiochromatograms are shown Figure S2)

### 3.3. In Vitro Characterisation

#### 3.3.1. Distribution Coefficient and Protein Binding

Log D of fluorophore conjugates showed a high dependence on the number of sulfate groups in their chemical structure. As expected, [^68^Ga]Ga-DAFC-Cy5 and ATTO 700 had a higher lipophilicity compared to the other dyes. Protein binding was quite high for [^68^Ga]Ga-DAFC-IRDye, whereas [^68^Ga]Ga-DAFC-Cy5, -SulfoCy7 and -ATTO 700 had low values around 10%. (Table 1)

#### 3.3.2. In Vitro Uptake of ^68^Ga-Siderophores

**Figure 2:**
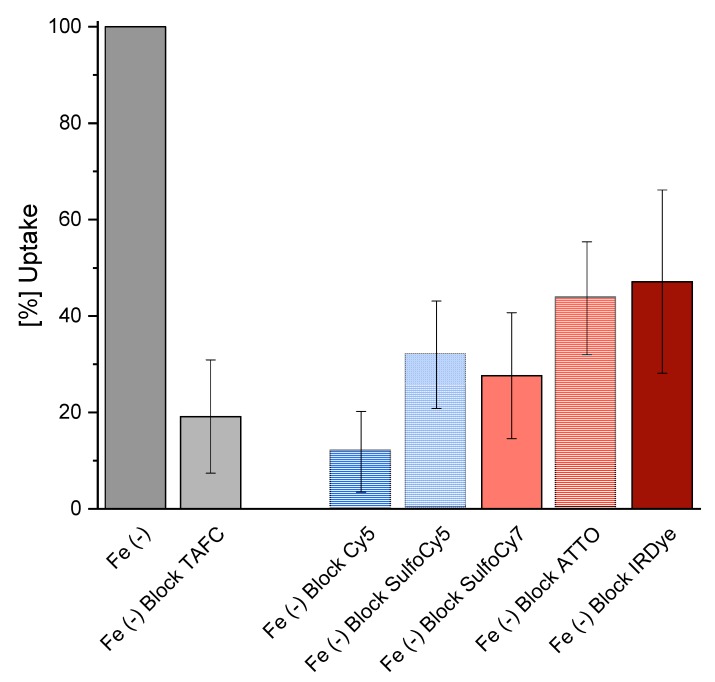
Competition assay of [^68^Ga]-Ga-TAFC blocked with [Fe]-Fluorophore compounds in iron depleted fungal culture [Fe (-)]. Reduction of [^68^Ga]Ga-TAFC uptake can be observed for all compounds, which indicates specific interaction with the MirB transporter.

The competition assay of the iron containing fluorophore conjugates resulted in a decrease of [^68^Ga]Ga-TAFC uptake for all compounds, respectively. (Figure 2) This indicates a competitive transport by the MirB transporter. Uptake values of the different radioactive labelled fluorophores are shown in the supplementary section (Figure S1).

#### 3.3.3. Utilization of Siderophore-Conjugates by *A. Fumigatus*

**Figure 3:**
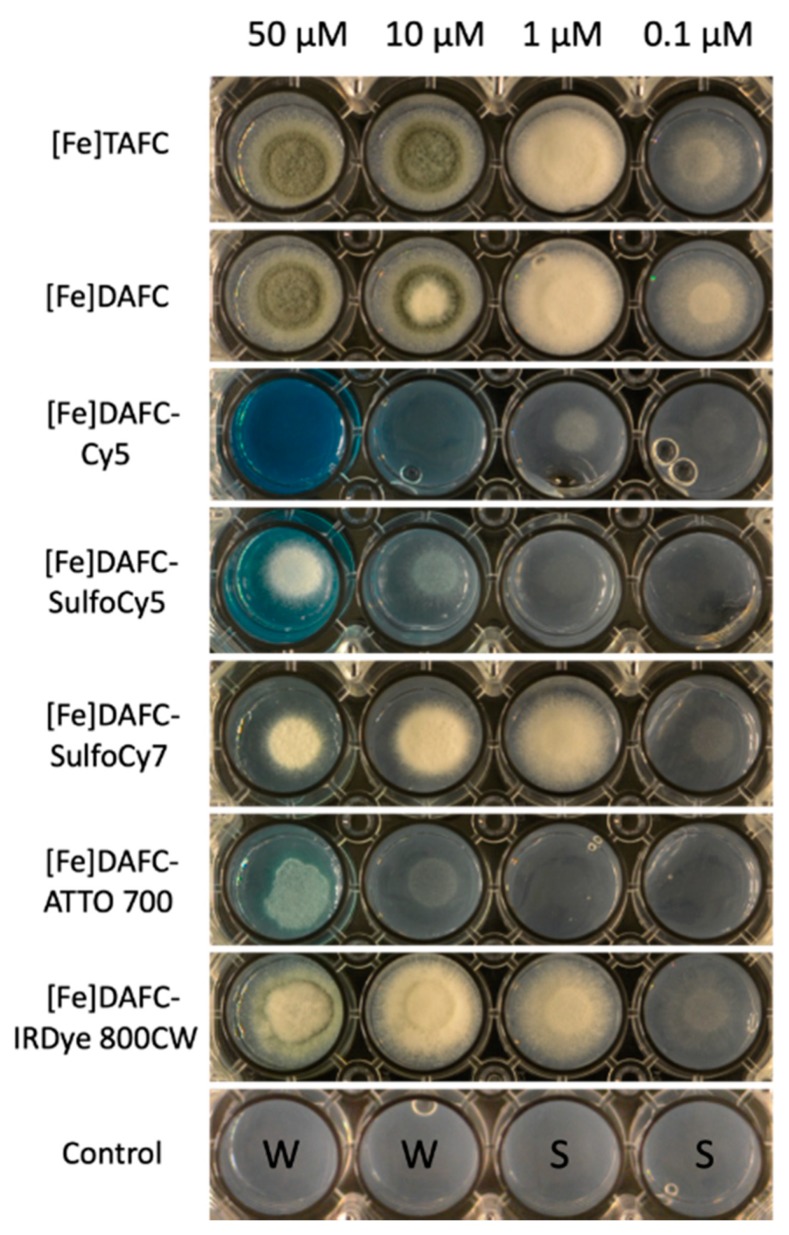
Growth of *A. fumigatus* mutant strain Δ*sidA/*Δ*ftrA* after 48 h incubation at 37 °C on iron-depleted Aspergillus minimal medium agar with different iron containing fluorophore conjugates. Growth is reflected by whitish-mycelia, while sporulation is reflected by the green colour, which arises from the green conidial-specific pigment. The last row shows controls of agar without siderophores: W = sterile water; S = Spores.

To measure utilization of the synthesized siderophore conjugates, a previously described bioassay was employed (14). Therefore, the *A. fumigatus* ΔsidA/ΔftrA mutant strain was used, which lacks both siderophore biosynthesis and reductive iron assimilation. Consequently, this strain is able to grow only in the presence of recognized siderophores or ferrous iron concentrations ≥3 mM. Control with [Fe]TAFC and [Fe]DAFC showed an induction of growth already at 0.1 µM and sporulation at 10 µM (Figure 3). [Fe]DAFC-IRDye supported growth to a slightly lower degree but did not support sporulation even at highest concentration. [Fe]DAFC-ATTO 700 weakly supported growth only at concentrations ≥10 µM. Interestingly, [Fe]DAFC-Cy5 supported growth at ≥1 µM but not with higher concentrations, indicating that a growth inhibitory effect occurred that arrested growth at ≥50 µM. [Fe]DAFC-SulfoCy7 supported growth to a similar degree as [Fe]DAFC-IRDye at 1 µM but showed a growth inhibitory effect at higher concentrations, as seen for [Fe]DAFC-Cy5, although not as severe.

#### 3.3.4. Fluorescence Microscopy

**Figure 4:**
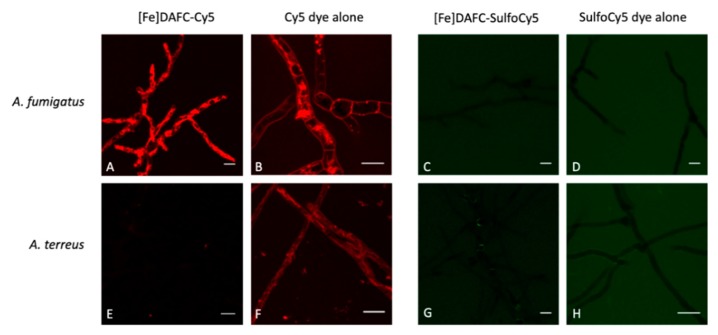
Fluorescence microscopy of [Fe]DAFC-Cy5 and [Fe]DAFC-SulfoCy5 in *A. fumigatus* and *A. terreus*. (**A**) [Fe]DAFC-Cy5 labels what appear to be tubular vacuoles with a clear concentration in hyphal tips. (**B**) Incubation with Cy5 carboxylic acid “dye alone” results in a very similar labelling pattern as [Fe]DAFC-Cy5. (**C**,**D**) In contrast, fluorescence microscopy does not visualize any uptake of [Fe]DAFC-SulfoCy5 and -SulfoCy5 “dye alone” by *A. fumigatus*. (**E**) *A. terreus*, which lacks a MirB homologous transporter and consequently TAFC uptake, does not show uptake of [Fe]DAFC-SulfoCy5, while it internalizes Cy5 carboxylic acid “dye alone” (**F**). (**G**,**H**) *A. terreus* does not internalize [Fe]DAFC-SulfoCy5 or -SulfoCy5 “dye alone”. Scale bars, 10 μm.

Fluorescence microscopy revealed a significant difference in the uptake of [Fe]DAFC-Cy5 and [Fe]DAFC-SulfoCy5 (Figure 4), which possess chemically similar structures (Figure 1). For [Fe]DAFC-Cy5, rapid uptake into hyphae of *A. fumigatus* with pronounced visualisation of subcellular organelles was observed 5 min after compound application, which remained visible for more than two hours. In contrast, no uptake of [Fe]DAFC-Cy5 was found by *A. terreus*, which lacks uptake of [Fe]TAFC (14). Remarkably, the Cy5 carboxylic acid dye alone showed rapid hyphal uptake in both *A. fumigatus* and *A. terreus*. These data demonstrate that the linking of DAFC imparts the feature of specific uptake by *A. fumigatus* to Cy5; [Fe]DAFC-Cy5 is specifically and actively imported, most likely by MirB, into hyphae of *A. fumigatus*.

In contrast, no uptake was observed for [Fe]DAFC-SulfoCy5 or the -SulfoCy5 carboxylic acid dye alone by *A. fumigatus* or *A. terreus* by fluorescence microscopy.

### 3.4. In Vivo Characterisation

#### µPET/CT and Ex Vivo Optical Imaging

**Figure 5:**
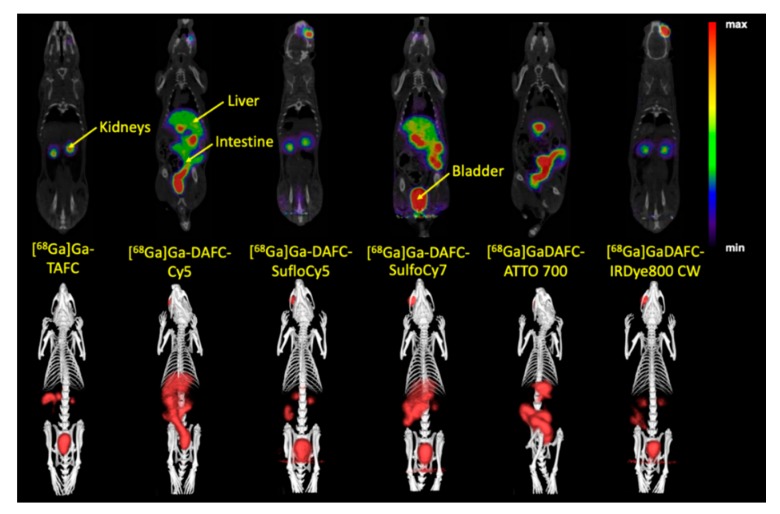
Coronal positron emission tomography (µPET/CT) slices (top row) and 3D volume rendered µPET/CT images (bottom row) of ^68^Ga-labelled fluorophore conjugates at 45 min p.i. in non-infected Lewis rats (approx. 5–10 MBq injected dose). Radioactive spots in the eye region originate from the retro-orbital injection.

µPET/CT images of non-infected Lewis rats injected with [^68^Ga]Ga-DAFC conjugated with various fluorophores revealed different biodistribution profiles (Figure 5). [^68^Ga]Ga-DAFC-Cy5, [^68^Ga]Ga-DAFC-SulfoCy7 and [^68^Ga]Ga-DAFC-ATTO 700 showed mainly excretion through the hepatobiliary route, [^68^Ga]Ga-DAFC-SulfoCy5 and [^68^Ga]Ga-DAFC-IRDye 800CW were excreted via the kidneys and bladder, similarly to [^68^Ga]Ga-TAFC.

**Figure 6:**
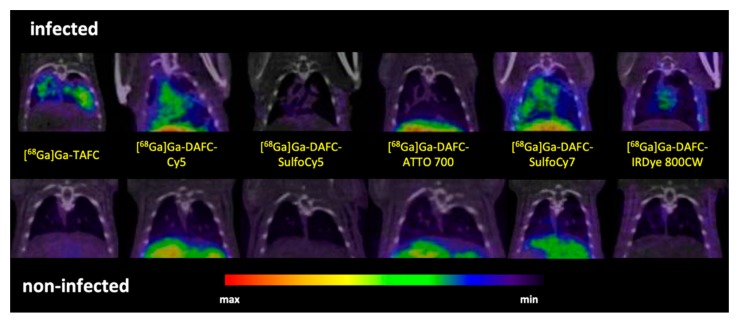
Coronal µPET/CT slices of *A. fumigatus* infected (top row) and non-infected animals (bottom row) 45 min p.i. in immunocompromised Lewis rats (approx. 5–10 MBq injected dose).

PET/CT imaging of *A. fumigatus* infected animals displayed clear accumulation of [^68^Ga]Ga-TAFC, [^68^Ga]Ga-DAFC-Cy5 and [^68^Ga]Ga-DAFC-SulfoCy7 in infected lungs. Certain uptake in the infected lung region was observed also for [^68^Ga]Ga-DAFC-IRDye 800CW, while [^68^Ga]Ga-DAFC-SulfoCy5 and [^68^Ga]Ga-DAFC-ATTO 700 imaging did not show any significant radioactive signal in the thoracic area (Figure 6).

**Figure 7:**
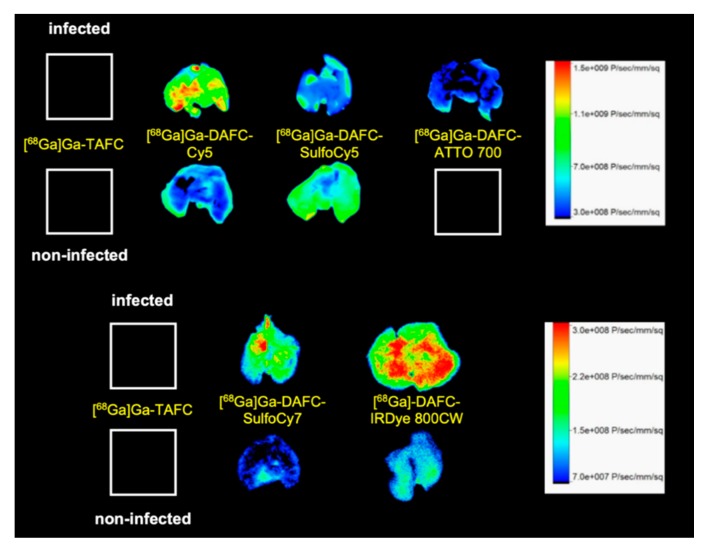
Fluorescence images of lungs excised from *A. fumigatus* infected and non-infected Lewis rats 1.5 h after the injection of different ^68^Ga-labelled fluorophore conjugates.

Optical imaging (Figure 7) of excised lungs revealed comparable results to PET/CT scans. [^68^Ga]Ga-DAFC-Cy5 displayed a high fluorescence signal in comparison to the non-infected control, even distributed over the whole organ. Both [^68^Ga]Ga-DAFC-SulfoCy7 and -IRDye 800CW showed also fluorescence in the infected lung tissue, however with slightly lower contrast to non-infected controls, whereas [^68^Ga]Ga-DAFC-SulfoCy5 and -ATTO 700 revealed a weak fluorescence signal in infected lungs and the fluorescence signal of [^68^Ga]Ga-DAFC-SulfoCy5 in non-infected lungs was even higher than in infected organ. [^68^Ga]Ga-TAFC displayed no fluorescence at all, which excludes an autofluorescence signal of the siderophore itself in the lungs.

## 4. Discussion

Hybrid imaging of fungal infections can be a very useful tool allowing not only diagnosis of IPA using PET/CT, but also precise localization of infected tissues during surgery or endoscopy by means of optical probes. Different hybrid imaging approaches have already been developed for oncological applications [25,26], but are currently very limited in the field of infection. First steps have been made to establish this method for bacterial infections [27] but so far this approach has not been investigated for fungal infections. Based on the sophisticated TAFC-dependent mechanism of *A. fumigatus* to acquire iron from the human host, the exchange of iron by gallium-68 resulted in successful PET imaging in an IPA rat model [14,16,28], the overall approach of using radiolabelled siderophores for molecular imaging is described in [29]. The specificity of [^68^Ga]Ga-TAFC for PET imaging of *A. fumigatus* has been shown by Petrik et al. in comparison with various organisms (fungi/bacteria) and also human lung cancer cells [24]. Doyle et al. modified [Fe]FsC with the fluorescent dye NBD (6-(*N*-(7-nitrobenz-2-oxa,1,3-diazol-4-yl)amino)Hexanoate) for fluorescent microscopy of *A. fumigatus* showing the feasibility of modifying siderophores retaining recognition by pathogenic fungi [30]. This study combines the advantages of both radioactive and fluorescent labelling in a hybrid imaging compound for fungal infection imaging. We had previously reported on the use of fusarinine C as a scaffold for hybrid imaging probes for tumor targeting, with [22] forming the basis for the modifications reported here.

Starting from [Fe]DAFC modification with different fluorophores using a HATU coupling strategy to activate carboxylic acid resulted in high yields in a very short time. After removing the iron from the complex, ^68^Ga-labelling in high radiochemical purity over 95% and high molar radioactivity was achieved for in vitro and in vivo experiments. Previous studies showed that different modifications, depending on size and charge of the functional groups, influence the uptake properties in *A. fumigatus* [14]. Fluorescent dyes used in this study were all charged from +1 to −3 with high molecular weight compared to the [Fe]DAFC molecule. This had a decisive influence on target interaction as well as pharmacokinetics. Competition assays showed blocking of [^68^Ga]Ga-TAFC uptake into hyphae indicating recognition of the conjugates by the MirB transporter. Even so, uptake assays resulted in a high unspecific binding, probably related to cell wall interactions due to charge and/or lipophilicity of the fluorophores. These findings could be clarified by employing growth assay using the *A. fumigatus* Δ*sidA/*Δ*ftrA* mutant strain, requiring external [Fe]siderophore supply for growth. Although there was a target interaction of all compounds, only [Fe]DAFC-Cy5, [Fe]DAFC-SulfoCy7 and [Fe]DAFC-IRDye 800CW promoted reasonable growth. Taken together, these data indicate that all conjugates interact with the MirB transporter but not all can be taken up by hyphae or, if transported, utilized for fungal iron metabolism. This was furthermore illustrated by fluorescence microscopy of [Fe]DAFC-Cy5 and [Fe]DAFC-SulfoCy5. [Fe]DAFC-Cy5 showed a fast internal accumulation into *A. fumigatus* hyphae with pronounced accumulation in apical organelles suspected to resemble tubular vacuoles [31] and/or mitochondria [32]. In contrast, no uptake at all was observed for [Fe]DAFC-SulfoCy5. MirB–dependence of [Fe]DAFC-SulfoCy5 was strongly indicated by the fact that *A. terreus*, which lacks MirB and [Fe]TAFC uptake. Ref. [14] also lacked uptake of [Fe]DAFC-SulfoCy5. In contrast to [Fe]DAFC-SulfoCy5, Cy5 carboxylic acid dye displayed non-specific uptake into both *A. fumigatus* and *A. terreus*, while both [Fe]DAFC-SulfoCy5 and -SulfoCy5 carboxylic acid did not accumulate in hyphae of *A. fumigatus* or *A. terreus*.

These results prove the specificity of the [Fe]DAFC-Cy5 conjugate for the MirB transporter. Previous studies used different siderophores, modified with fluorescent dyes, for example desferroxamine (DFO) with cypate [33] or ferrichrome with naphthalimide or quantum dots to visualize cellular structures [34]. However, these compounds have only been used for microscopy and not for hybrid imaging applications.

In vivo µPET/CT images of the conjugates in an IPA rat model confirmed in vitro data. As expected, excretion of each compound could be connected to the Log D and protein binding values. Lipophilic compounds like [^68^Ga]Ga-DAFC-Cy5/ATTO 700 or [^68^Ga]Ga-DAFC-SulfoCy7 showed mainly hepatobiliar excretion, whereas hydrophilic conjugates [^68^Ga]Ga-DAFC-SulfoCy5/IRDye 800CW were primarily eliminated through the kidneys. In line with the rapid uptake of [Fe]DAFC-Cy5 in microscopy studies, visualization of the infected lung area was also clearly observed for [^68^Ga]Ga-DAFC-Cy5. Moreover, lack of accumulation of [^68^Ga]Ga-DAFC-SulfoCy5 in the IPA model corresponded to growth assays and microscopy.

To complete these findings, ex vivo optical images of lungs showed the same pattern as µPET/CT scans. Except for [^68^Ga]Ga-DAFC-SulfoCy5, where a slightly higher signal in the non-infected control was detected, all other infected lungs showed a more intense signal than respective non-infected controls.

Overall, our results show that functional modification of DAFC as a mimic for the *A. fumigatus* specific siderophore TAFC is possible, and that the introduction of fluorescent dyes provides reliable hybrid imaging compounds for IPA. Recognition by the target and pharmacokinetics were highly dependent on the fluorophore applied. Furthermore, in addition to novel hybrid imaging applications, these compounds also provide insight into the structure related target interaction and uptake characteristics to design potentially antifungal siderophore conjugates.

## 5. Conclusions

Overall, this study shows that the modification of TAFC with various fluorescent dyes is possible and they are still recognized by *A. fumigatus*. Radiolabelling is easily achievable with high stability in vivo and in vitro. This work reveals insight into the structure related properties of modified siderophores, especially with respect to their combination with antifungal drugs and species-specific fluorescence microscopy applications. This opens up new possibilities for applications to combine PET with optical imaging (e.g., surgery) as a hybrid imaging agent in IPA with *A. fumigatus*.

## Figures and Tables

**Table 1:** Distribution coefficient and protein binding of siderophore compounds radiolabelled with gallium-68.

		DAFC-Cy5	DAFC-SulfoCy5	DAFC-SulfoCy7	DAFC-ATTO 700	DAFC-IRDye 800CW
Distribution coefficient	Log D (pH 7.4)	1.03 ± 0.105	−3.38 ± 0.100	−2.19 ± 0.068	−0.81 ± 0.032	−2.65 ± 0.042
*n* = 3		*n* = 18	*n* = 18	*n* = 18	*n* = 18	*n* = 19
Protein binding [%] *n* = 3	30 min	13.7 ± 2.9	5.3 ± 0.6	14.9 ± 2.2	6.9 ± 0.5	57.1 ± 3.8
	60 min	13.1 ± 2.3	5.3 ± 0.7	18.9 ± 0.8	8.8 ± 0.8	58.1 ± 3.7
	120 min	13.7 ± 1.8	7.3 ± 1.1	22.6 ± 1.8	11.7 ± 1.4	54.8 ± 2.7

Data are presented as mean ± SD.

## Supplementary Materials

The following are available online at https://www.mdpi.com/2218-273X/10/2/168/s1, Figure S1: Uptake of different radiolabelled siderophores normalized on [^68^Ga]Ga-TAFC. Figure S2: Representative radio-HPLC chromatograms of ^68^Ga-labelled siderophore compounds.
