# Supplementary material for: Hybrid Imaging of Aspergillus fumigatus Pulmonary Infection with Fluorescent, 68Ga-Labelled Siderophores
Source: Biomolecules. 2020 Jan 22;10(2):168. doi: 10.3390/biom10020168 (PMC7072563; doi:10.3390/biom10020168)
Supplement: Supplementary file 1 [file biomolecules-10-00168-s001.pdf]

Supplement

Analytical RP-HPLC: Reversed-phase (RP) high-performance Liquid chromatography (HPLC) analysis was carried out using the following instrumentation: UltiMate 3000 RS UHPLC pump, UltiMate 3000 autosampler, UltiMate 3000 Variable Wavelenght Detector; UV detection at λ = 220nm (Dionex, Germering, Germany) Radio-detector (Gabi Star, Raytest; Straubenhardt, Germany) using Jupiter 5 µM C_{18} 300 Å 150 x 4.6 mm (Phenomenex Ltd. Aschaffenburg, Germany) as a column with acetonitrile (ACN)/H_2O/0.1% trifluoroacetic acid (TFA) as mobile phase; flow rate of 1 mL/min;

Gradient A: 0.0–1.0 min 10% ACN, 1.0–12.0 min 10–60 % ACN, 13.0–15.0 min 60–80 % ACN, 15.0–16.0 min 80–10% ACN, 16.0–20.0 min 10% ACN.

Gradient B: 0.0–1.0 min 30% ACN, 1.0–12.0 min 30–80 % ACN, 13.0–15.0 min 80–100 % ACN, 15.0–16.0 min 100–30% ACN, 16.0–20.0 min 30% ACN.

Gradient C: 0.0–3.0 min 10 % ACN, 3.0–20.8 min 10–100 % ACN, 16.8–24.0 min. 100 % ACN, 24.1-27.0 min 10 % ACN.

Preparative RP-HPLC. Sample purification via RP-HPLC was carried out on a Gilson 322 Pump with a Gilson UV/VIS-155 detector (UV detection at λ = 220 nm) using a PrepFC™ automatic fraction collector (Gilson, Middleton, WI, USA), Eurosil Bioselect Vertex Plus 30 x 8 mm 5 µm C_{18}A 300Å pre-column and Eurosil Bioselect Vertex Plus 300 x 8 mm 5 µm C_{18}A 300Å column (Knauer, Berlin, Germany) and the following ACN/H_2O/ 0.1 % TFA gradients with a flow rate of 2 mL/min:[18]

Gradient 1: 0.0–5.0 min 0 % ACN, 5.0–35.0 min 0–50 % ACN, 35.0–38.0 min 50 % ACN, 38.0–40.0 min 50–0 % ACN,

Gradient 2: 0.0–1.0 min 10 % ACN, 1.0–35.0 min 10–60 % ACN, 35.0–36.0 min 60 % ACN, 36.0–38.0 min. 60–10 % ACN,

Gradient 3: 0.0–1.0 min 20 % ACN, 1.0–35.0 min 20–80 % ACN, 35.0–38.0 min 100 % ACN, 38.0–40.0 min. 100–20 % ACN,

Gradient 4: 0.0-1.0 min 10 % ACN, 1.0-35.0 min 10-60 % ACN, 35.0-40.0 min 60 % ACN, 40.0-41.0 min 60-80 % ACN, 41.0-45.0 min 80 % ACN, 45.0-46.0 min 80-10 % ACN.

MALDI-TOF MS: Matrix-assisted laser desorption/ionization time -of-flight mass spectrometry was performed on a Bruker microflex™ bench-top MALDI-TOF MS (Bruker Daltonics, Bremen, Germany). Samples were prepared on a micorscout target (MSP96 target ground steel BC, Bruker Daltonics) using dried-droplet method and α-cyano-4-hydroxycinnamic acid (HCCA, Sigma-Aldrich, Handels GmbH, Vienna, Austria) as matrix. All spectra were recorded by summarizing 600 laser shots per spot and Flex Analysis 2.4 software was used for data processing.

Precursor Preparation:

[Fe]Fusarinin C ([Fe]FsC): Fusarinin C was obtained by fungal culture according to Schrettl *et al* [8]. *Aspergillus fumigatus* mutant strain ΔSidG (which lacks the enzyme for acetylation of Fusarinin C) was seeded ($1*10^6$ Spores/mL) in 200 mL iron depleted minimal medium, incubated for 28h at 37°C and shaking at 200 rpm. After filtering of the culture supernatant, $FeSO_4$ was added to a final concentration of 10mM to get a red coloured solution. The filtrate was subsequently loaded to a Reveleris silica flash cartridge (C18; 40µm; 12g; column volume (CV) of 18mL; BÜCHI Labortechnik AG, Flawil, Switzerland) by using a REGLO tubing pump (Type ISM795, Ismatec SA, Glattbrugg-Zürich, Switzerland) with a flow rate of 10 mL/min. Fixed [Fe]FsC on the cartridge was washed with 2

CV of water and then eluted with 5 CV of methanol. After evaporation of the organic solvent, approximately 70 mg of [Fe]FsC could be obtained as a red-brown coloured solid with a purity of >90% confirmed by analytical RP-HPLC (gradient A t_R = 6.68 min), and the product was used for synthesis without further purification. MALDI-TOF-MS: *m/z* [M+H] = 780.68 [$C_{33}H_{51}FeN_6O_{12}$; exact mass: 779.63 (calculated)]

Acetylation of [Fe]Fusarinin C:

To acetylate [Fe]FsC 30 mg (38 µmol) dissolved in 500 µL water was rocked with 20 µL (0.2 µmol) of acetic anhydride for 2 min at room temperature and intense shaking. Resulting products mono-, di-, and triacetylfusarinin C were immediately purified via preparative RP-HPLC using gradient 1 to collect N,N′-diacetylfusarinine C ([Fe]DAFC, t_R = 20.3 min) in high purity (> 95%) followed by lyophilization. MALDI-TOF-MS: *m/z* [M+H] = 864.01 [$C_{37}H_{55}FeN_6O_{14}$; exact mass: 863.70 (calculated)]. [20]

Conjugation of Fluorophores

Conjugation of Sulphated Cyanine Dyes:

[Fe]DAFC (5.0 mg, 5.8 µmol) was dissolved in anhydrous DMF and 1.1 equivalent of Sulfo-Cyanine 5-NHS ester (Lumiprobe GmbH, Hannover, Germany) dissolved in dry DMF was added and, after pH adjustment (pH 8.5) with DIPEA, the reaction mixture was stirred at RT under light exclusion for three hours. After quantitative conjugation, the reaction solution was purified by RP-HPLC (gradient 2, t_R = 24.8 min) to give a dark blue coloured solid after lyophilisation. Analytical data: [Fe]DAFC-SulfoCy 5 5.2 mg [3.49 µmol, 60 %]; RP-HPLC gradient A, t_R = 9.51 min; MALDI TOF-MS: m/z [M+H] = 1488.73 [$C_{69}H_{91}FeN_8O_{21}S_2$; exact mass: 1488.48 (calculated)].

For the conjugation of SulfoCyanine 7-carboxylic acid (Lumiprobe GmbH, Hannover, Germany), the fluorescent dye (2.0 mg, 2.7 µmol) was dissolved in dry DMF and, after the addition of 2 equivalents HATU (2.0 mg, 5.2 µmol), the mixture was kept at ambient temperature under gentle shaking to activate the carboxylic acid. After 10 min, 1 equivalent [Fe]DAFC (2.3 mg, 2.7 µmol) dissolved in dry DMF was added, pH 8 to 9 was adjusted by adding DIPEA and the reaction was stirred for two hours at RT. [Fe]DAFC-SulfoCy 7 was purified by preparative RP-HPLC (gradient 2, t_R = 28.3 min) and freeze dried to give a dark green powder. Analytical data: [Fe]DAFC-SulfoCy7 3.5 mg [2.26 µmol, 83 %]; RP-HPLC gradient A, t_R = 10.49 min; MALDI TOF-MS: m/z [M+H] = 1555.49 [$C_{74}H_{97}FeN_8O_{21}S_2$; exact mass: 1554.58 (calculated)].

For the conjugation of IRDye 800CW carboxylic acid (2 mg, 1.83 µmol; LI-Core, Lincoln, Nebraska USA), the fluorescent dye was dissolved in dry DMF and, after addition of 2 equivalents HATU, the mixture was kept at ambient temperature under gentle shaking to activate the carboxylic acid. After 10 min, 1 equivalent [Fe]DAFC dissolved in dry DMF was added, pH 8 to 9 was adjusted by adding DIPEA and the reaction was stirred for two hours at RT. The conjugates were purified by preparative RP-HPLC (gradient 2, [Fe]DAFC-IRDye 800CW t_R = 22.3 min) and freeze dried to give a dark green powder. Analytical data: [Fe]DAFC-IRDye 800CW 2.6 mg [1.44 µmol, 79 %]; RP-HPLC gradient A, t_R = 8.7 min; MALDI TOF-MS: m/z [M+H]+ = 1848.30 [$C_{84}H_{109}FeN_7O_{28}S_4$; exact mass: 1848.89 (calculated)].

Conjugation of Non-Sulphated Cyanine Dyes:

Cyanine 5-carboxylic acid (1.0 mg, 1.93 µmol, Lumiprobe GmbH, Hannover, Germany) was dissolved in 1 mL DMF and, after addition of 2 equivalents HATU (1.47 mg), the reaction mixture was stirred for 15 min at RT. Thereafter, 2 equivalents of [Fe]DAFC (3.33 mg) and 20 µL DIPEA were added and the resulting mixture was left to react at RT. After one hour, the reaction solution was purified via preparative RP-HPLC (gradient 3; t_R = 27.8 min) to give the dark blue coloured product after freeze drying. Analytical data: [Fe]DAFC-Cy5 2.1 mg [1.58 µmol, 84 %]; RP-HPLC gradient B; t_R = 10.47 min; MALDI TOF-MS: m/z [M+H]+ = 1329.88 [$C_{69}H_{92}FeN_8O_{15}$; exact mass: 1329.36 (calculated)].

Conjugation of Xanthene Based Dyes

Activation of ATTO 700-carboxylic acid (1.0 mg, 1.74 µmol, ATTO-TEC GmbH, Stegen, Germany) was done with 2 equivalents HATU (1.32 mg) in 500 µl DMF under stirring at RT for 10 min. Following this, 2 equivalents [Fe]DAFC were dissolved in 500 µl DMF and pH 9 was adjusted with DIPEA. In the next step, both solutions were combined and reaction took place at RT for 1.5 h. Finally, the product was purified using RP-HPLC (gradient 4; t_R = 30.0 min) and lyophilization to appear as a dark green coloured powder. Analytical data: [Fe]DAFC-ATTO 700 1.80 mg [1.28 µmol, 74 %]; RP-HPLC gradient B; t_R = 11.45 min; MALDI TOF-MS: m/z [M+H$^+$] = 1411.23; [$C_{68}H_{90}FeN_8O_{19}S$; exact mass: 1411.39 (calculated)].

Demetallation

For the purpose of iron removal, the corresponding conjugates were dissolved in 1–2 mL H_2O/organic solvent (e.g. ACN, DMF, EtOH) 20% (v/v) and 1–2 mL of aqueous Na_2EDTA solution (100mM) was added. The resulting mixtures were stirred under light exclusion for 4 h at ambient temperature followed by preparative RP-HPLC purification to give the iron free fluorescent conjugates after lyophilisation.

DAFC-Cy5: 0.9 mg [blue, 0.71 µmol, 78 %], gradient 3 (t_R = 28.3); Analytical data: RP-HPLC gradient B; t_R = 10.75 min; MALDI TOF-MS: m/z [M+H] = 1277.06 [$C_{69}H_{95}N_8O_{15}$; exact mass: 1276.53 (calculated)].

DAFC-SulfoCy5: 2.8 mg [blue, 1.94 µmol, 72 %], gradient 2 (t_R = 25.2 min); Analytical data: RP-HPLC gradient A, t_R = 9.77 min; MALDI TOF-MS: m/z [M+H] = 1435.87 [$C_{69}H_{94}N_8O_{21}S_2$; exact mass: 1435.66 (calculated)];

DAFC-SulfoCy7: 1.4 mg [green, 0.92 µmol, 72 %] gradient 2 (t_R = 28.9 min); Analytical data: RP-HPLC gradient A, t_R = 10.67 min; MALDI TOF-MS: m/z [M+H] = 1502.53 [$C_{74}H_{100}N_8O_{21}S_2$; exact mass: 1501.77 (calculated)].

DAFC-ATTO700: 0.5 mg [green, 0.38 µmol, 42 %] gradient 4 (t_R = 34.0 min); Analytical data: RP-HPLC gradient B, t_R = 11.74 min; MALDI TOF-MS: m/z [M+H] = 1358.45 [$C_{68}H_{93}N_8O_{19}S$; exact mass: 1358.57 (calculated)]

DAFC-IRDye800CW: 1.2 mg [green, 0.65 µmol, 90 %] gradient 2 (t_R = 23.0 min); Analytical data: RP-HPLC gradient A, t_R = 8.9 min; MALDI TOF-MS: m/z [M+H]+ = 1796.10 [$C_{84}H_{112}N_7O_{28}S_4$; exact mass: 1796.07 (calculated)].

In vitro uptake of ^{68}Ga-Siderophores:

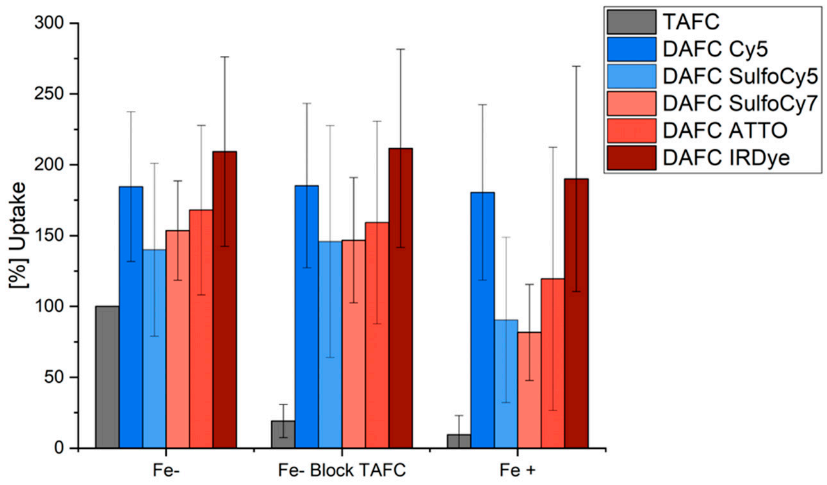

Figure S1. Uptake of different radiolabelled siderophores normalized on [^{68}Ga]Ga-TAFC. High uptake of the fluorophore compounds indicate an unspecific binding to the cell surface of the hyphae. In addition, blocking with [Fe]TAFC resulted in no significant reduction of compound accumulation. In iron replete conditions, uptake was reduced but still had a high unspecific binding to the fungal culture.

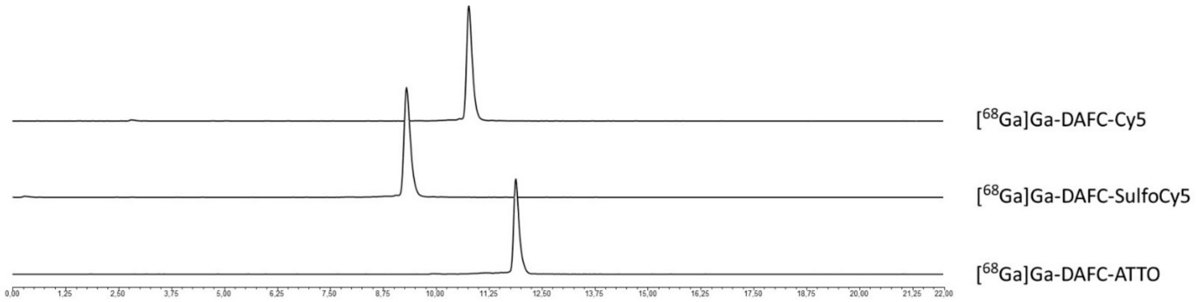

Figure S2. Representative radio-HPLC chromatograms of ^{68}Ga-labelled siderophore compounds.
